# Local Arginase Inhibition during Early Reperfusion Mediates Cardioprotection via Increased Nitric Oxide Production

**DOI:** 10.1371/journal.pone.0042038

**Published:** 2012-07-31

**Authors:** Adrian T. Gonon, Christian Jung, Abram Katz, Håkan Westerblad, Alexey Shemyakin, Per-Ove Sjöquist, Jon O. Lundberg, John Pernow

**Affiliations:** 1 Divison of Clinical Physiology, Department of Laboratory Medicine, Karolinska Institutet, Karolinska University Hospital, Stockholm, Sweden; 2 Divison of Cardiology, Department of Medicine, Karolinska Institutet, Karolinska University Hospital, Stockholm, Sweden; 3 Department of Physiology and Pharmacology, Karolinska Institutet, Karolinska University Hospital, Stockholm, Sweden; 4 Department of Bioscience, AstraZeneca, Mölndal, Sweden; University of Pecs Medical School, Hungary

## Abstract

Consumption of L-arginine contributes to reduced bioavailability of nitric oxide (NO) that is critical for the development of ischemia-reperfusion injury. The aim of the study was to determine myocardial arginase expression and activity in ischemic-reperfusion myocardium and whether local inhibition of arginase within the ischemic myocardium results in increased NO production and protection against myocardial ischemia-reperfusion. Anesthetized pigs were subjected to coronary artery occlusion for 40 min followed by 4 h reperfusion. The pigs were randomized to intracoronary infusion of vehicle (n = 7), the arginase inhibitor N-hydroxy-nor-L-arginine (nor-NOHA, 2 mg/min, n = 7), the combination of nor-NOHA and the NO synthase inhibitor N^G^-monomethyl-L-arginine (L-NMMA, 0.35 mg/min, n = 6) into the jeopardized myocardial area or systemic intravenous infusion of nor-NOHA (2 mg/min, n = 5) at the end of ischemia and start of reperfusion. The infarct size of the vehicle group was 80±4% of the area at risk. Intracoronary nor-NOHA reduced infarct size to 46±5% (P<0.01). Co-administration of L-NMMA abrogated the cardioprotective effect mediated by nor-NOHA (infarct size 72±6%). Intravenous nor-NOHA did not reduce infarct size. Arginase I and II were expressed in cardiomyocytes, endothelial, smooth muscle and poylmorphonuclear cells. There was no difference in cytosolic arginase I or mitochondrial arginase II expression between ischemic-reperfused and non-ischemic myocardium. Arginase activity increased 2-fold in the ischemic-reperfused myocardium in comparison with non-ischemic myocardium. In conclusion, ischemia-reperfusion increases arginase activity without affecting cytosolic arginase I or mitochondrial arginase II expression. Local arginase inhibition during early reperfusion reduces infarct size via a mechanism that is dependent on increased bioavailability of NO.

## Introduction

Rapid restoration of coronary blood flow by means of either primary percutaneous intervention or thrombolysis is standard treatment for patients with acute ST-elevation myocardial infarction [1]. However, acute reperfusion of the jeopardized myocardium results in a cascade of harmful events, referred to as reperfusion injury. Factors contributing to reperfusion injury include endothelial and microvascular dysfunction, activation of pro-inflammatory cascades, generation of free oxygen radicals and intracellular calcium overload [2], [3]. A central feature of reperfusion injury is reduced bioavailability of nitric oxide (NO) [4]. Strategies to increase the availability of NO include the substrate L-arginine or NO donors which have been shown to confer protection against ischemia and reperfusion injury [5], [6]. NO bioavailability may be critically regulated by arginase by competing with NO synthase (NOS) for their common substrate L-arginine [7]. Arginase, which converts L-arginine to ornithine and urea, has been demonstrated to be upregulated in the vasculature in several conditions associated with endothelial dysfunction including atherosclerosis [8], diabetes mellitus [7], [9] and during liver and myocardial ischemia-reperfusion [10], [11]. Two distinct isoforms of arginase (I and II) have been described. Arginase I is a cytosolic enzyme expressed primarily in the liver but also in vascular smooth muscle, endothelial cells and the myocardium [12], [13]. Arginase II is a mitochondrial enzyme expressed in various tissues [14], [15], [16] including the heart and the vasculature [12].

Consumption of L-arginine by upregulation of arginase may result in reduced bioavailability of NO during myocardial ischemia-reperfusion and could thereby contribute to ischemia-reperfusion injury. Blockade of arginase might be a promising strategy to improve the availability of L-arginine for the production of NO leading to diminished ischemia-reperfusion injury. We recently demonstrated that systemic inhibition of arginase significantly reduced infarct size in a rat model of ischemia-reperfusion [11]. However, since the arginase inhibitor was given iv before the onset of ischemia, it is not known whether the effect was due to inhibition of liver arginase and subsequent increase in systemic nitrite levels which in turn may be reduced to NO [6], or if it had a local effect in the myocardium at the time of reperfusion. Furthermore, the effect of ischemia-reperfusion on myocardial arginase activity in relation to protein expression remains to be evaluated. The aim of the present study was therefore to investigate whether local myocardial arginase activity contributes to ischemia-reperfusion injury and whether such an effect is dependent on interference with the local production of NO. This was tested in a clinically relevant model using local infusion of an arginase inhibitor before start of reperfusion.

## Materials and Methods

### Ethics Statement

The study was approved by the regional ethical committee for animal experiments (Swedish Board of Agriculture, Norra Djurförsöksetiska Nämnd, approval number N 324/07) and conforms to the Guide for Care and Use of Laboratory Animals published by the US National Institutes of Health (NIH publication No. 85-23, revised 1996).

### Surgical Procedures

Twenty-five female farm pigs (27–38 kg) were premedicated with a combination of tiletamin (1.5 mg/kg im), zolezepam (1.5 mg/kg im) and medetomidin hydrochloride (0.06 mg/kg im). Anaesthesia was initially induced by injection of sodium pentobarbital (20 mg/kg iv) and maintained with sodium pentobarbital (2–4 mg kg/h iv) and morphine (0,5 mg/kg/h iv). The animals received heparin 5000 IU/h iv. The animals were intubated and mechanically ventilated with air and oxygen. Respiratory rate and tidal volume were adjusted to keep arterial blood pH, pO_2_ and pCO_2_ within the physiological range. Rectal temperature was kept at 39.0±0.2°C by means of a heated operating table. A central venous catheter was inserted in the right external jugular vein for drug and fluid administration. Another catheter was positioned in the descending aorta via the right femoral artery for sampling of blood and for measurement of arterial pressure via a pressure transducer. Heart rate was determined from the arterial pressure curve. All variables were continuously recorded on personal computer equipped with PharmLab V3.0 (AstraZeneca R&D, Mölndal, Sweden). The heart was exposed via a sternotomy. A ligature was placed around the left anterior descending artery (LAD) at a position from which the distal third of the artery is occluded when tightening the ligature. A thin needle connected to a catheter was placed in the LAD distal to the ligature for intracoronary (ic) administration of the experimental drugs during ischemia and reperfusion into the jeopardized area. An ultrasonic probe (Transonic Systems Inc., New York, USA) was placed around the artery just proximal to the snare for measurement of coronary blood flow. The flow probe was connected to a Transonic 208 blood flow meter.

**Figure 1:**
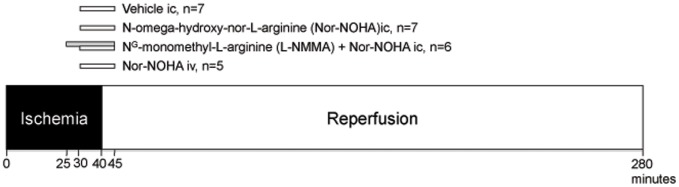
Experimental protocols. The animals were subjected to 40 min of ischemia and 240 min reperfusion. The groups were randomized to vehicle ic, nor-NOHA ic, nor-NOHA combined with L-NMMA ic or nor-NOHA iv. All infusions were started at 30 min of ischemia except L-NMMA which was started at 25 min of ischemia. The infusions were maintained until 5 min of reperfusion.

### Experimental Protocol

After a post-surgery stabilization period of 30 min the pigs were subjected to myocardial ischemia induced by tightening the ligature around the LAD. The animals were randomized to receive ic infusion of either 1) saline (0.9% NaCl, vehicle, n = 8), 2) the arginase inhibitor N-omega-hydroxy-nor-L-arginine (2.0 mg/min, nor-NOHA, Bachem, Bubendorf, Switzerland, n = 8) or 3) the NO synthase (NOS) inhibitor N^G^-monomethyl-L-arginine (0.35 mg/min, L-NMMA, Alexis Biochemicals, Lausen, Switzerland, n = 6) together with nor-NOHA. A fourth group received nor-NOHA 2 mg/min as a systemic iv infusion (n = 5). In addition a fifth group of sham operated pigs (n = 5) was added to be used as a control group for the analysis of arginase I and II and arginase activity. Two pigs (one pig randomized to the vehicle group and one in the nor-NOHA group) were excluded from the study due to irreversible ventricular fibrillation occurring during ischemia.

As depicted in Fig. 1, the infusions of vehicle or nor-NOHA were started at 30 min of ischemia and continued up to 5 min after start of reperfusion. The infusion of L-NMMA was started at 25 min of ischemia and continued until 5 min after initiation of reperfusion. The dose of L-NMMA was based on earlier studies in our laboratory [17]. All infusions were given at a rate of 2 ml/min. After 40 min of ischemia LAD was reperfused for 4 h by removal of the ligature. At the end of reperfusion the LAD was reoccluded and 1 mg/kg of 2% Evans Blue (Sigma, St Louis, MO, USA) was injected iv to outline the area at risk, after which the animals were sacrificed by an iv injection of potassium chloride. The heart was rapidly extirpated. The atria and the right ventricle were removed. The left ventricle was cut into 1 cm thick slices perpendicular to the heart base-apex axis. Myocardial pieces of the third slice from the apex were used for expression analyses of arginase I and II using immunohistochemistry and Western blotting. The remaining myocardial slices were placed in 0.8% triphenyltetrazolium chloride (Sigma, St Louis, MO, USA) at 37°C for 10 min which stained viable myocardium red. The extent of area risk and myocardial necrosis were determined by planimetry using Adobe PhotoshopC5. Five ml of blood was sampled from the abdominal aorta before ischemia and at 5, 20, and 60 min of reperfusion for determination of nitrite.

Additional sham pigs (n = 5) were anaesthetized and surgery was performed as described above but the animals were not subjected to myocardial ischemia. Myocardial tissue samples were collected for subsequent immunohistochemistry and protein expression.

**Table 1:** Haemodynamic variables before ischemia and during reperfusion.

	Pre-isch			60 min rep				120 min rep			180 min rep			240 min rep		
**Vehicle ic**																
Heart rate (bpm)	93±9			109±5				116±9			133±10			135±19		
MAP (mmHg)	95±6			70±6				62±6			62±5			52±7		
LAD flow (ml/min)	20.1±1.8			24.6±6.8				18.0±4.1			16.9±3.7			8.8±2.1		
RPP (bpm×mmHg)	9014±1466			7800±822				7106±862			8197±894			7482±1321		
**Nor-NOHA ic**																
Heart rate (bpm)	97±7			121±5				117±3			120±6			125±10		
MAP (mmHg)	95±4			71±4				67±5			62±4			56±5		
LAD flow (ml/min)	17.0±1.9			25.6±7.4				19.9±2.8			14.1±2.6			9.9±1.7		
RPP (bpm×mmHg)	9175±750			8555±589				7897±567			7301±329			6768±621		
**L-NMMA + nor-NOHA ic**																
Heart rate (bpm)	79±6			106±7				109±9			116±11			117±10		
MAP (mmHg)	97±5			72±4				72±5			65±6			60±4		
LAD flow (ml/min)	15.7±1.3			21.3±6.2				22.4±3.2			14.8±1.5			10.1±0.7		
RPP (bpm×mmHg)	7737±794			7747±848				7922±835			7507±924			7001±780		
**Nor-NOHA iv**																
Heart rate (bpm)	105±8			105±8				110±7			117±9			115±5		
MAP (mmHg)	96±5			65±5				67±7			65±7			55±8		
LAD flow (ml/min)	17.4±2.1			17.0±4.8				16.7±5.6			11.1±2.0			5.6±0.8		
RPP (bpm×mmHg)	9985±505			6704±388				7473±1123			7567±908			6313±884		

Abbreviations: MAP, mean arterial pressure; LAD, left anterior descending coronary artery; RPP rate pressure product (RPP); ic, intracoronary; iv, intravenous. Data are presented as mean±SEM. There were no significant differences between the groups.

### Preparation of Cytosolic and Mitochondrial Fractions

Preparation of mitochondrial fractions was based on a method described elsewhere [18]. Briefly, frozen pieces of pig heart were transferred to 10 volumes of ice-cold buffer consisting of (in mM): sucrose, 250; KCl, 20; EDTA, 1; and Hepes, 5 (pH 7.4). After thawing, the muscles were minced while in the buffer with cold scissors and then homogenized with a loose fitting motor-driven Teflon pestle in a glass homogenizor. Homogenization was performed at low speed on ice for 2 min (15 s on, 5 s off). Samples were then centrifuged at 2°C for 10 min at 750× g. The pellet was discarded and the supernatant was centrifuged at 2°C for 10 min at 12000× g. The supernatant was used as the cytosolic fraction and the pellet was resuspended (2 µl/mg wet wt) in a buffer consisting of (in mM): Hepes, 20; NaCl, 150; EDTA, 5; KF, 25; Na_3_VO_4_, 1; 20% glycerol; 0.5% TritonX, and protease inhibitor cocktail (Roche, Basel, Switzerland) 1 tablet per 50 ml, and this represented the mitochondrial fraction. Fractions were frozen at −80°C until processed. In frozen muscle, there is considerable leakage of matrix proteins into the cytosol, as judged by measurements of citrate synthase activity (data not shown). However, proteins associated with mitochondrial membranes remain intact, as judged by Western blots of the fractions for cytochrome oxidase where strong bands are seen in the mitochondrial fractions and weak or no bands are seen in the cytosolic fractions.

### Western Blots

Cytosolic and mitochondrial fractions were diluted with reduced Laemmli buffer and heated at 70°C for 10 min. Fifteen (arginase I) or 5 µg (arginase II) of protein were loaded onto each well and separated by electrophoresis using NuPAGE Novex 4–12% Bis-Tris Gels (Invitrogen) and transferred onto polyvinylidine fluoride membranes (Immobilon FL, Millipore). Membranes were blocked for 1 h at room temperature in Li-Cor Blocking buffer (LI-COR Biosciences), followed by incubation overnight at 4°C with the following primary antibodies diluted in blocking buffer: mouse anti-dihydropyridine receptor (DHPR; 1∶500, #ab2864, Abcam), mouse anti-oxphos complex IV subunit I (cytochrome oxidase, 1∶1000, #459600, Invitrogen), rabbit anti-arginase I (1∶2000, #HPA003595, Sigma), and rabbit anti-arginase II (1∶2000, #HPA000663, Sigma). Membranes were then washed in TBS-T and incubated for 1 h at room temperature with IRDye 680-conjugated goat anti-mouse IgG and IRDye 800-conjugated goat anti-rabbit IgG (1∶15,000, LI-COR) blocking buffer and 0.01% SDS. Membranes were then washed in TBS-T and imunoreactive bands visualized using infrared fluorescence (IR-Odyssey scanner, LI-COR Biosciences). Band densities were analysed with Image J (NIH, USA; http://rsb.info.nih.gov/ij/).

**Figure 2:**
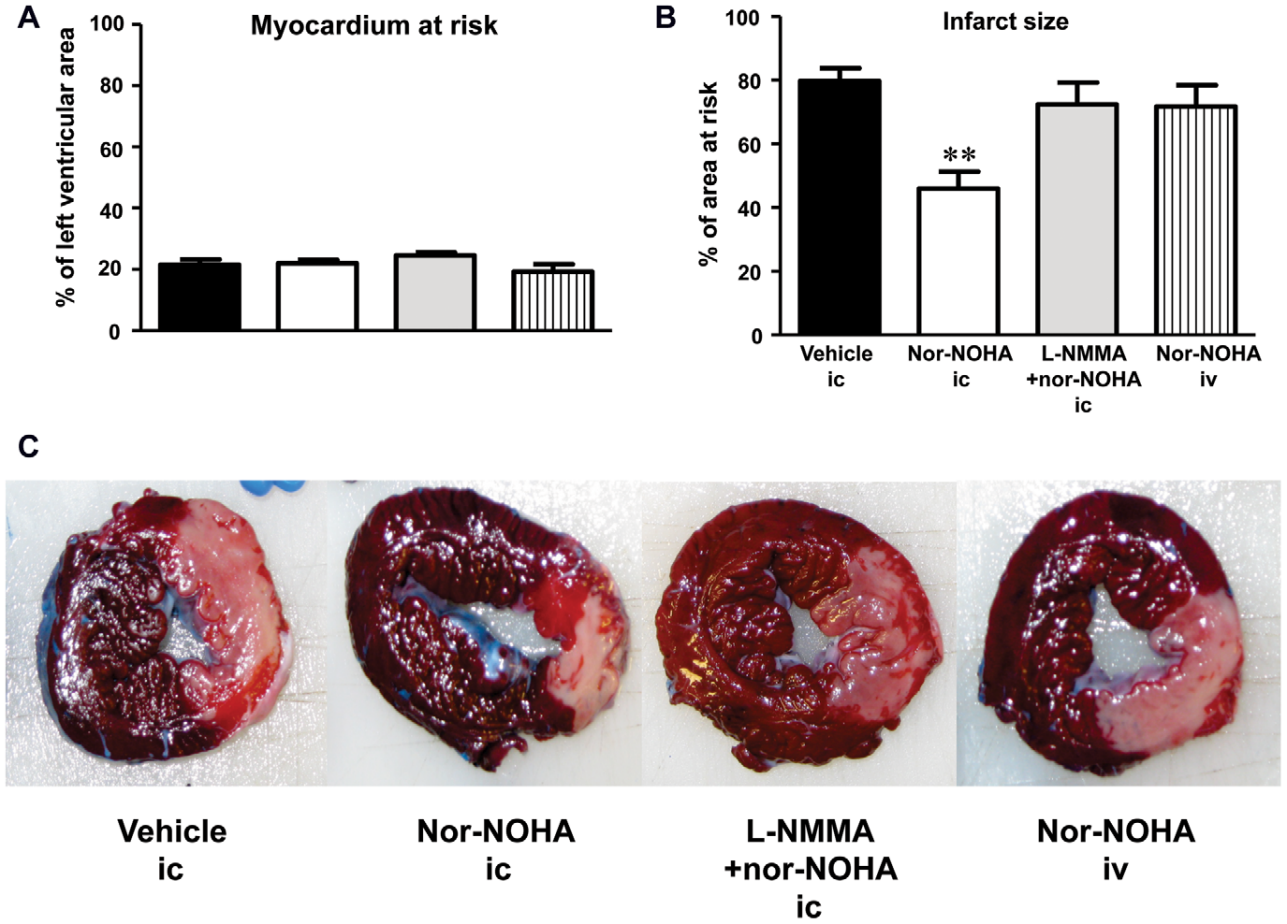
Area at risk (A), infarct size (B) and representative triphenyltetrazolium chloride stained cross section of the left ventricle (C) of the groups given ic vehicle, the arginase inhibitor nor-NOHA, the combination of the NOS-inhibitor L-NMMA and nor-NOHA, or iv administration of nor-NOHA. Mean and SEM; n = 5–7. Significant difference from the vehicle group is indicated; **P<0.01.

### Determination of Nitrite

Blood was centrifuged immediately after sampling at 1200 g for 10 min at 4°C and stored at −80°C until analysis. Nitrite was measured using a highly sensitive chemiluminescence method described in detail before [19].

**Figure 3:**
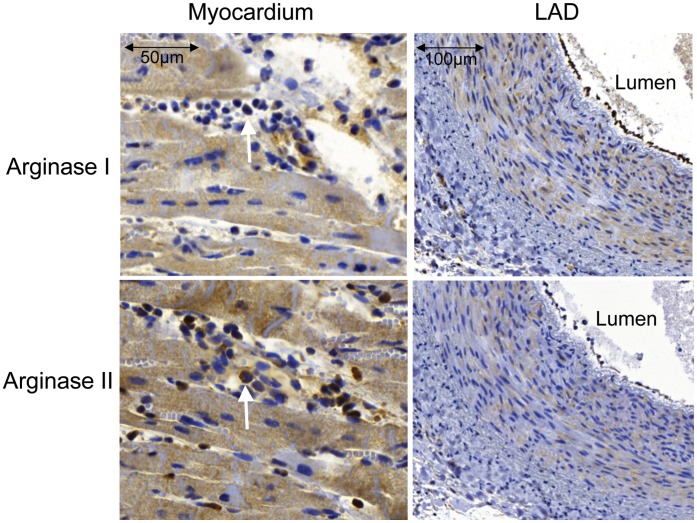
Immunohistochemical staining demonstrating the expression of arginase I (upper panel) and arginase II (lower panel) in ischemic-reperfused myocardium (left panel) and left anterior descending artery (right panel). Both isoforms are expressed in cardiomyocytes, polymorphonuclear cells (arrows), vascular smooth muscle cells and endothelial cells.

### Arginase Activity Assay

Arginase activity was determined by using a colorometric assay previously described by Berkowitz et al. [20]. The assay measures the urea content using α-isonitrosopropiophenone. To detect arginase activity only the inhibitable fraction of urea was used in the analysis. A lysis buffer consisting of PBS, 1 mM EDTA, Triton X (0.1%) and protease inhibitors (Roche) was freshly prepared. Lysates of homogenized myocardial tissue were centrifuged for 15 min at 14000 g at 4°C. 50 µl of the supernatant were added to 75 µl of Tris-HCl (50 mM, pH 7.5) containing 10 mM MnCl_2_. The mixture was activated by heating for 10 min at 56°C. Each sample was then incubated at 37°C for 1 h under three conditions: (1) with L-arginine (50 µl, 0.05 M, in Tris-HCl pH 9.7), (2) with only Tris-HCl (50 µl, pH 9.7) and (3) with L-arginine (50 µl, 0.05 m, in Tris-HCl pH 9.7) and 30 min preincubation with the arginase inhibitor 2(S)-amino-6-boronohexanoic acid (ABH; 100 µM; Enzo Clinical Labs, Farmingdale, NY, USA). The reaction was stopped by adding 400 µl of an acid solution (H_2_SO_4_–H_3_PO_4_–H_2_O = 1∶3:7). 25 ul of α-isonitrosopropiophenone (9% in ethanol) was added to each sample, and the mixture was then heated at 100°C for 60 min. The samples were placed in the dark for 10 min and the urea concentration was determined at 550 nm using spectrophotometry. The urea inhibitable fraction was then calculated and used in statistical analyses.

### Statistical Analysis

All values are presented as mean±S.E.M. Two group comparisons were analyzed by Mann-Whiney U test. Multiple comparisons were made by Kruskal Wallis followed by Dunńs test. The level of significance was set at P<0.05.

**Figure 4:**
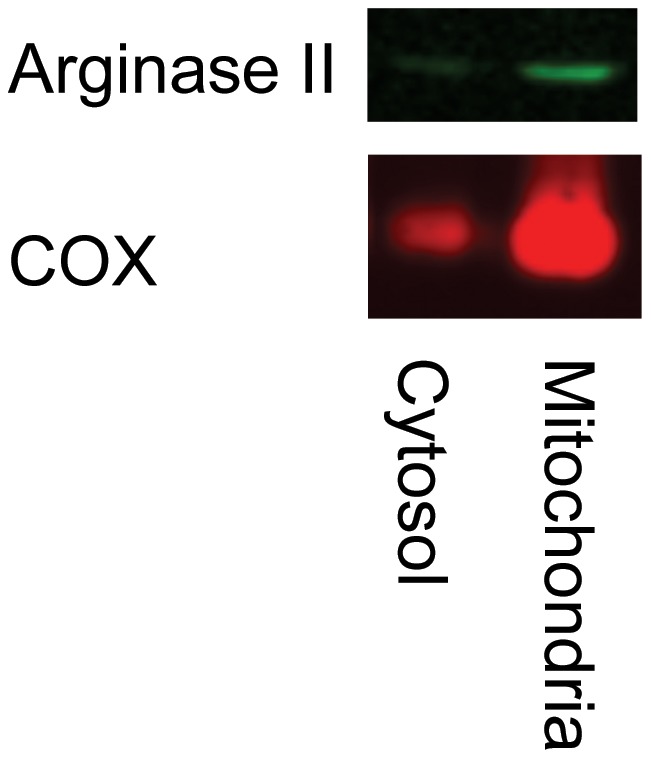
Expression of arginase II in the cytosolic and mitochondrial fractions of a representative myocardial sample. For comparison the expression of the mitochondrial membrane protein cytochrome oxidase (COX) is depicted.

**Figure 5:**
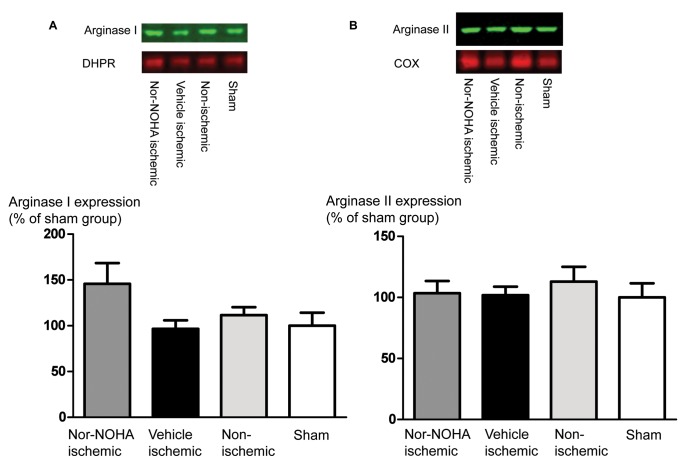
Representative blots (upper panel) and quantitative analysis of the expression (lower panel) of arginase I in the cytosolic fraction (A) and arginase II in the mitochondrial fraction (B) of myocardial samples from the non-ischemic and ischemic area of pigs subjected to ischemia-reperfusion and myocardium from sham operated pigs. Expression of arginase I is normalized to that of the surface membrane L-type Ca^2+^channel, dihydropyridine receptor (DHPR) and arginase II to that of cytochrome oxidase (COX) and presented in % of the mean expression in the sham group. Mean±SEM, n = 6. There were no significant differences between the groups.

## Results

### Hemodynamics

A total number of eight pigs developed ventricular fibrillation during 15–30 min of ischemia. Six of them randomized to vehicle (n = 3), nor-NOHA (n = 2) and the combination of nor-NOHA and L-NMMA (n = 1) were successfully converted to sinus rythm by DC countershocks of 20–30 J. The remaining two pigs (one pig randomized to vehicle and one to nor-NOHA group) developed irreversible ventricular fibrillation before drug administration and were excluded from the study. The hemodynamic changes during the experiments are shown in Table 1. There were no statistically significant differences in heart rate, mean arterial pressure, rate pressure product or LAD blood flow between the groups.

### Area at Risk and Infarct size

Figure 2 depicts the area at risk and infarct size. There were no significant differences in area at risk between the groups (Fig. 2A). The infarct size was significantly reduced in the group given ic infusion of the arginase inhibitor nor-NOHA in comparison with the vehicle group (46±5% vs. 80±4% of the area at risk, P<0.01, Fig. 2B). Systemic iv administration of the same dose of nor-NOHA did not affect infarct size (72±7% of the area at risk). Co-administration with the NOS inhibitor L-NMMA abrogated the cardioprotection mediated by ic nor-NOHA (infarct size 72±7%, Fig. 2B). Representative stained myocardial sections are depicted in Fig. 2C.

### Plasma Nitrite

Arterial plasma nitrite concentration did not change significantly following administration of nor-NOHA. Plasma nitrite was 680±160 nM before the onset of ischemia, 692±116 nM 5 min after the onset of reperfusion (during nor-NOHA infusion) and 681±119 nM at 60 min of reperfusion. Furthermore, arterial plasma nitrite was unchanged in the vehicle group.

### Immunohistochemical Analysis of Arginase Expression

As illustrated in Fig. 3 arginase I and II expression were detected in cardiomyocytes and in intramural coronary arteries of ischemic-reperfused and sham hearts. Polymorphonuclear cells present in ischemic-reperfused myocardium expressed both arginase I and II. In addition, arginase I and II were expressed in endothelial cells and vascular smooth muscle cells of LAD collected from ischemic-reperfused and sham hearts.

### Western Blots

Separation of mitochondrial membrane fractions was confirmed by expression of cytochrome oxidase in the mitochondrial fraction and only weak or no expression in the cytosolic fraction (Fig. 4). The cytosolic and mitochondrial fractions of non-ischemic, ischemic-reperfused and sham operated myocardium were analyzed for arginase I and II expression. Expression of arginase I in the cytosol was similar in all groups (Fig. 5A), whereas arginase II was not expressed in the cytosolic fraction (e.g. Fig. 4 and data not shown). Arginase II was clearly expressed in the mitochondrial fraction, but there were no significant differences between the ischemic-reperfused, non-ischemic or sham myocardium (Fig. 5B).

### Arginase Activity in the Myocardium

**Figure 6:**
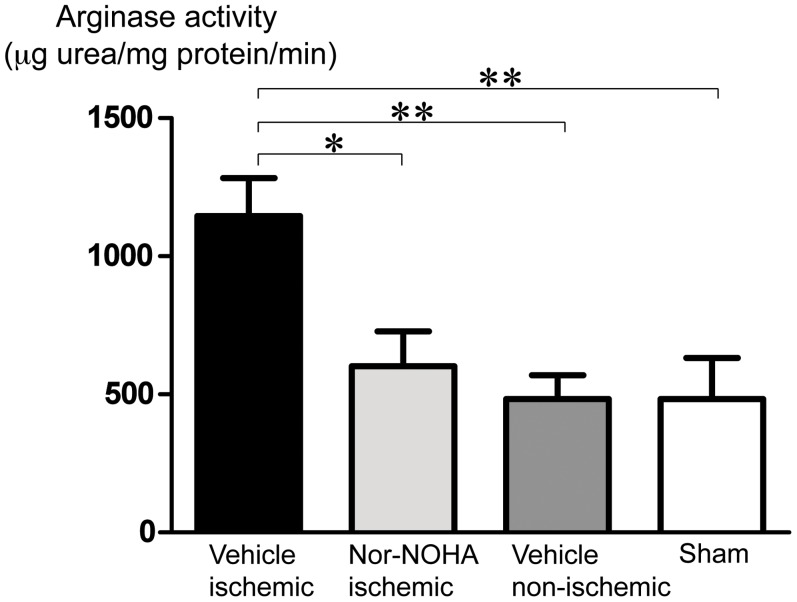
Arginase activity in myocardial samples from ischemic and non-ischemic myocardium of pigs subjected to ischemia-reperfusion and myocardium from sham operated pigs. Mean±SEM, n = 6–7. *P<0.05, **P<0.01.

The activity of arginase was similar in the myocardium of sham operated pigs and in the non-ischemic myocardium from pigs subjected to ischemia-reperfusion. Arginase activity was 2-fold higher in ischemic-reperfused myocardium than in non-ischemic myocardium of pigs given vehicle and in comparison with the sham group (P<0.01, Fig. 6). Nor-NOHA administration during early reperfusion inhibited significantly arginase activity in the ischemic-reperfused myocardium in comparison with the vehicle group (P<0.05, Fig. 6).

## Discussion

The main findings of this study are 1) that ischemia and reperfusion up-regulates arginase activity in the ischemic myocardium, and 2) that local ic administration of the arginase inhibitor nor-NOHA into the vessel supplying the risk area during early reperfusion markedly reduced infarct size. The cardioprotective effect of nor-NOHA was abrogated by the NOS inhibitor L-NMMA indicating that the effect of arginase blockade was mediated via increased production of NO.

The pathophysiology behind ischemia-reperfusion injury is complex. Previous studies have demonstrated a crucial role of maintained bioavailability of NO to protect from ischemia-reperfusion injury [5], [21]. Upregulation of arginase that competes with NOS for their common substrate L-arginine may be an important mechanism by which NO bioavailability is reduced during ischemia-reperfusion. The functional importance of arginase was investigated by ic infusion of the arginase inhibitor nor-NOHA starting before the onset of reperfusion. This resulted in reduction of infarct size by 50%. A previous study demonstrated that iv administration of an arginase inhibitor before the onset of ischemia protects from ischemia-reperfusion injury [11] but this is the first study demonstrating that local myocardial arginase is an important factor contributing to ischemia-reperfusion injury. The observation that the same dose of nor-NOHA given systemically did not affect infarct size supports the notion that the effect is mediated via a local action within the myocardium at risk and not via systemic arginase inhibition. This conclusion is further supported by the observation that arterial nitrite levels were unaffected by nor-NOHA. This is of importance since previous studies have shown that systemic arginase inhibition increases plasma nitrite [11] and that increases in systemic plasma nitrite levels by only 30% considerably reduced infarct size in mice [6]. The cardioprotective effect of nor-NOHA in the present study was obtained by ic infusion during late ischemia and early reperfusion. This observation, which indicates that the treatment protected from reperfusion injury rather than having an anti-ischemic effect, is of clinical interest since the protocol with short-term administration of nor-NOHA during early reperfusion may be feasible in the clinical setting in connection with primary coronary angioplasty.

The mechanism behind the local cardioprotective effect of nor-NOHA was investigated by local ic co-administration with the NOS inhibitor L-NMMA. The cardioprotective effect of nor-NOHA was completely prevented by NOS blockade demonstrating that the infarct sparing effect of arginase blockade is dependent on intact NOS activity in the jeopardized myocardium.

To investigate the molecular mechanisms behind the functional effect of arginase inhibition during ischemia-reperfusion, arginase expression and activity were determined. It was found that ischemia-reperfusion is associated with increased arginase activity. Detailed analysis of protein expression revealed that arginase I and arginase II protein levels in cytosolic and mitochondrial fractions, respectively, were unaffected by ischemia-reperfusion. This observations supports the notion that the increased activity may be independent of changes in protein levels and instead may be due to post-translational modification. One possibility is increased activity of arginase I by S-nitrosylation [22], [23]. However, S-nitrosylation was shown to be dependent on inducible NOS which does not seem to be up-regulated in the myocardium in the present experimental model [24]. Therefore, the exact mechanism behind increased arginase activity during ischemia-reperfusion remains to be determined. Immunohistochemical analysis revealed that both arginase I and II are expressed in several cell types including cardiomyocytes and vascular endothelial and smooth muscle cells of both ischemic-reperfused and non-ischemic myocardium. There was no evident difference in the localization of arginase between ischemic-reperfused and non-ischemic myocardium. An interesting observation is that arginase was expressed in polymorphonuclear cells associated with ischemia-reperfusion injury [25]. From the present data it cannot be determined which cell type that contributes to the functional involvement of arginase in terms of increased activity or ischemia-reperfusion injury. The finding that the cardioprotective effect of nor-NOHA was dependent on NO production may indicate that endothelial cell arginase is of functional importance.

There are limitations with the present study that deserve consideration. Since both arginase I and II are expressed and nor-NOHA inhibits both isoforms we cannot determine whether one or both isoform is of functional importance in the setting of ischemia-reperfusion. This issue needs to be further evaluated using isoform specific inhibitors when such are available. Further, it is not known which NOS isoform is involved since L-NMMA blocks all three enzymes (NOS1, 2 and 3). However, it is unlikely that NOS2 plays a crucial role due to the short experimental protocol. It has earlier been demonstrated that cardioprotection mediated via interference with NOS is not associated with changes in NOS2 expression in the presently used experimental model [24].

In conclusion, the present study demonstrates that myocardial ischemia-reperfusion increases arginase activity without affecting cytosolic arginase I or mitochondrial arginase II expression, and that inhibition of local myocardial arginase protects the heart against ischemia-reperfusion injury via increased production of NO. These effects are achieved by local ic infusion of an arginase inhibitor during late ischemia and early reperfusion suggesting that such treatment would be feasible for patients undergoing primary percutaneous angioplasty for ST elevation myocardial infarction.
